# A Novel, Cardiac-Derived Algorithm for Uterine Activity Monitoring in a Wearable Remote Device

**DOI:** 10.3389/fbioe.2022.933612

**Published:** 2022-07-19

**Authors:** Muhammad Mhajna, Boaz Sadeh, Simcha Yagel, Christof Sohn, Nadav Schwartz, Steven Warsof, Yael Zahar, Amit Reches

**Affiliations:** ^1^ Nuvo-Group, Ltd, Tel-Aviv, Israel; ^2^ Department of Obstetrics and Gynecology, Hadassah Medical Center, Faculty of Medicine, Hebrew University of Jerusalem, Jerusalem, Israel; ^3^ Department of Obstetrics and Gynecology, University Hospital, Heidelberg, Germany; ^4^ Maternal and Child Health Research Center, Perelman School of Medicine, University of Pennsylvania, Philadelphia, PA, United States; ^5^ Ob-Gyn/MFM at Eastern Virginia Medical School, Norfolk, VA, United States

**Keywords:** biomedical signal processing, electrocardiography (ECG), Gynecology & Obstetrics, phonocardiography (PCG), telemedicine, wearable device, uterine activity, uterine contractions

## Abstract

**Background:** Uterine activity (UA) monitoring is an essential element of pregnancy management. The gold-standard intrauterine pressure catheter (IUPC) is invasive and requires ruptured membranes, while the standard-of-care, external tocodynamometry (TOCO)’s accuracy is hampered by obesity, maternal movements, and belt positioning. There is an urgent need to develop telehealth tools enabling patients to remotely access care. Here, we describe and demonstrate a novel algorithm enabling remote, non-invasive detection and monitoring of UA by analyzing the modulation of the maternal electrocardiographic and phonocardiographic signals. The algorithm was designed and implemented as part of a wireless, FDA-cleared device designed for remote pregnancy monitoring. Two separate prospective, comparative, open-label, multi-center studies were conducted to test this algorithm.

**Methods:** In the intrapartum study, 41 laboring women were simultaneously monitored with IUPC and the remote pregnancy monitoring device. Ten patients were also monitored with TOCO. In the antepartum study, 147 pregnant women were simultaneously monitored with TOCO and the remote pregnancy monitoring device.

**Results:** In the intrapartum study, the remote pregnancy monitoring device and TOCO had sensitivities of 89.8 and 38.5%, respectively, and false discovery rates (FDRs) of 8.6 and 1.9%, respectively. In the antepartum study, a direct comparison of the remote pregnancy monitoring device to TOCO yielded a sensitivity of 94% and FDR of 31.1%. This high FDR is likely related to the low sensitivity of TOCO.

**Conclusion:** UA monitoring via the new algorithm embedded in the remote pregnancy monitoring device is accurate and reliable and more precise than TOCO standard of care. Together with the previously reported remote fetal heart rate monitoring capabilities, this novel method for UA detection expands the remote pregnancy monitoring device’s capabilities to include surveillance, such as non-stress tests, greatly benefiting women and providers seeking telehealth solutions for pregnancy care.

## 1 Introduction

Uterine activity (UA) monitoring is one of the crucial measurements for antepartum fetal monitoring and intrapartum labor surveillance as well as for the detection of preterm labor. Uterine contractions need to be evaluated to monitor the progress of labor (Ayres-De-Campos et al., 2015). Additionally, their correlation to the fetal heart rate provides important information on fetal well-being during both antepartum and intrapartum stages (Bakker and Van Geijn, 2008; Nageotte, 2015; Warmerdam et al., 2016).

Monitoring UA is performed by several techniques. The gold-standard intrauterine pressure catheter (IUPC) involves transvaginal insertion of a catheter into the uterus. This can only be performed by an experienced obstetrician after the rupture of membranes and sufficient cervical dilation, limiting its use to a small percent of laboring patients, with no application to outpatient antenatal care. Tocodynamometry (TOCO), the non-invasive standard-of-care method, utilizes an external strain-gauge transducer positioned on the maternal fundus to measure deformations of the maternal abdomen due to uterine contractions (Bakker et al., 2007). Therefore, TOCO is significantly influenced by the quality of the skin–transducer interface, which is affected by misalignment of the transducer, the tension of the belt securing the transducer, maternal movement, and the BMI of the pregnant woman (Vlemminx et al., 2018). Failure to overcome these challenges had led to a reduced ability of TOCO to register uterine contractions, with a sensitivity as low as 46–74% relative to IUPC (Nguyen et al., 2016)– (Hayes-Gill et al., 2012).

*Electrohysterography (EHG)*, a promising alternative for monitoring UA, has been recently evaluated for its increased sensitivity to uterine contractions (86–95% compared with the standard (Jacod et al., 2010; Hayes-Gill et al., 2012; Euliano et al., 2013; Hadar et al., 2015; Nguyen et al., 2016; Vlemminx et al., 2017)), and for its improved performance in high BMI patients compared to TOCO (Vlemminx et al., 2018; Euliano et al., 2013). EHG utilizes multiple electrodes to record the electrical activity of the uterine muscle, which is assumed to be correlated with uterine contractions (Steer and Hertsch, 1950). However, EHG was reported to detect a higher number of contractions, some of which were considered false, relative to both IUPC and TOCO (Vlemminx et al., 2017; Hadar et al., 2015; Cohen and Hayes-Gill, 2014). Moreover, several technical challenges, such as electrode positioning (Alberola-Rubio et al., 2013); Marchon et al., 2018), interelectrode distance (Rooijakkers et al., 2014), electrical interference, and skin preparation (Tam and Webster, 1977), need to be addressed before this method can be widely adopted.

Recently, there is a growing interest in telehealth, especially in light of the COVID-19 pandemic making in-office visits difficult (Aziz et al., 2020; Nakagawa et al., 2020). Remote monitoring of pregnancy is considered beneficial to both the pregnant woman and healthcare providers. Women who are physically limited from coming to the clinic will gain improved accessibility to obstetrical services; and better clinical outcomes are expected, such as identifying and preventing preterm labors and other conditions that require early diagnosis and treatments (DeNicola et al., 2020; Lanssens et al., 2017; Xie et al., 2020). Additional potential benefits of remote monitoring include cost reduction through decreased antepartum hospitalization time and improved neonatal outcomes (Buysee et al., 2008; Barbour et al., 2017; Lanssens et al., 2018; Butler Tobah et al., 2019). Specifically, the ability to perform remote non-stress tests (NSTs) could be of great benefit to women and providers seeking telehealth solutions for pregnancy care. Conducting remote NSTs is a challenging task since it requires self-application of a highly accurate, non-invasive, and reliable device.

To date, all common techniques for externally monitoring UA are not able to be self-administered, either because they are designed for use in a healthcare setting under the direct supervision of medical personnel, or their core technology is not easily self-administered. Some of these remote monitoring systems capture uterine activity utilizing TOCO (Van Den Heuvel et al., 2020;Van Den Heuvel et al., 2019) and others use EHG (Euliano et al., 2013;Jacod et al., 2010;Devedeux et al., 1993); however, EHG-based devices often require single-use adhesive sensors and are currently approved only for term pregnancies (FDA approved K140862 and K153262). A new method for monitoring UA could effectively address these challenges.

INVU™ (Nuvo Group Ltd.) is a physician-prescribed remote pregnancy monitoring system comprising a sensor band that houses a set of biopotential sensors, acoustic sensors, and motion sensors. These sensors, placed on the maternal abdomen, accurately and continuously acquire the maternal and fetal cardiovascular signals. We have previously demonstrated the ability of INVU to remotely obtain accurate fetal and maternal heart rates (FHR and MHR) (Mhajna et al., 2020).

**FIGURE 1:**
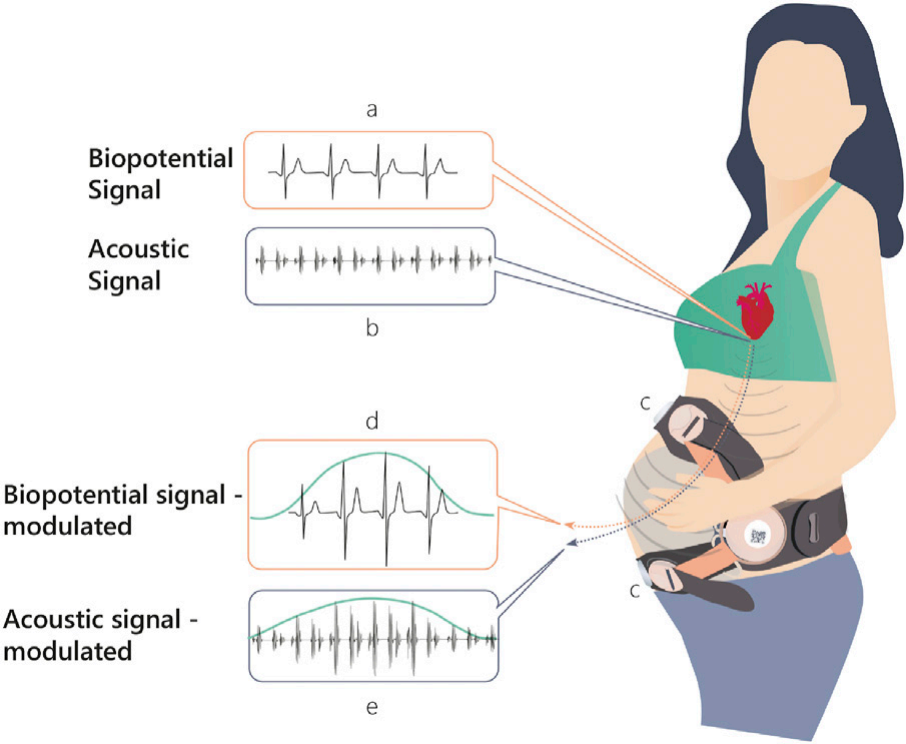
Physiological mechanism of action upon which INVU’s cardiac-based uterine monitoring (CaBUM) algorithm is built.

Here, we present a novel solution for monitoring UA. Figure 1 shows the basic mechanism of action behind the method. During uterine contractions, the structure of the myometrium at both the cellular level and the organ level changes significantly, altering the internal body media. Consequently, the propagation of the electrical (Figure 1A) and acoustic signals (Figure 1B) generated by the maternal and fetal hearts are altered, leading to detectable changes in their mapping on the maternal abdominal surface (Figure 1C). The mapping of these signals, both electrical (electrocardiogram [ECG])and acoustic (phonocardiogram [PCG]), is modulated by the uterine contractions (Figures 1D,E). The recorded raw signals from the abdominal surface sensors include not only the maternal ECG and PCG signals but they also include noise sources originating from other maternal organs such as muscles (EMG), stomach (acoustic), lungs (both acoustic and electrical noises), or noise originating from the fetus, fetal ECG, and PCG signals. Moreover, both maternal and fetal movement may introduce noise to the recorded signals. Other external noise sources like powerline interference or external sounds may also impact the recorded raw signals. All these noises should be treated properly in the pre-processing stage. The study presents an innovative algorithm for monitoring UA focusing on the technical aspects of the algorithm while presenting case studies and statistical results.

The cardiac-based uterine monitoring (CaBUM) algorithm has been recently FDA cleared (K210025), allowing the INVU system to fully perform remote NSTs. Moreover, the compromise of reducing the quality of the aforementioned FHR and MHR recordings due to the conflicting nature of optimal sensor positions for UA and FMHR monitoring (Rooijakkers et al., 2014) is avoided with the INVU by using a single sensor system for both measurements. The clinical performance of non-invasively detecting UA with INVU while conducting remote NSTs has been recently described and validated (Schwartz et al., 2022).

## 2 Materials and Methods

### 2.1 Overall Framework of the Wearable Monitoring Belt

**FIGURE 2:**
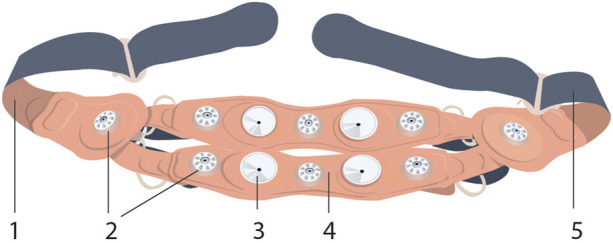
INVU sensor band™ inner-side view (the side facing the abdominal skin) is shown, detailing the (1 and 5) rear-closing buckle; (2) electrocardiogram sensors, eight in total; (3) acoustic sensors, four in total; and (4) textile band (this figure was published in N. Schwartz et al., “Novel Uterine Contraction Monitoring to Enable Remote, Self-administered Non-stress Testing,” Am. J. Obstet. Gynecol., 2021)

**FIGURE 3:**
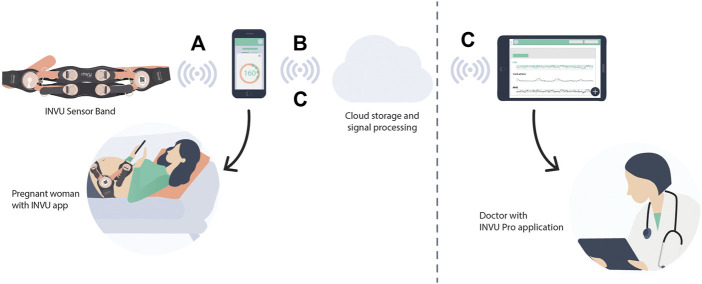
System diagram. **(A)** Biopotential and acoustic signals are acquired by the sensors in the band and are transformed via Bluetooth to a nearby mobile device that had already been paired with the INVU device. **(B)** Data are then transmitted wirelessly and securely via WiFi from the mobile device to the Cloud Application. The signals are processed at the cloud server level, including signal processing to identify fetal and maternal cardiac signals and uterine contractions, and the results are downloaded in real time to the mobile devices of the pregnant woman and her medical team via a web-based application **(C)**.

The INVU wearable belt collects sensory data using two types of sensors: biopotential sensors that acquire the body’s electrical activities, and acoustic sensors that acquire sounds originating from within the pregnant woman’s abdomen. The acoustic sensors are highly sensitive microphones that transduce sound waves into an analog electrical signal. The biopotential sensors measure small potential or voltage changes on the skin that arise from physiological signals, including the cardiac electrical signals generated during each heartbeat. Details of the components of the INVU sensor band are illustrated in Figure 2. Analog data from each sensor are sent to an analog-to-digital (A/D) conversion module, which samples the analog signals and sends packets by Bluetooth to a mobile device, which in turn transmits the signal wirelessly and securely via WiFi to the cloud for processing. The cloud receives the data from the application and performs the proprietary signal processing to identify fetal and maternal cardiac signals and uterine contractions. The processed data are then sent via a web-based application to the mobile devices of the pregnant woman and her healthcare provider. Figure 3 illustrates the entire system data flow.

### 2.2 Hardware Circuit and Device Design

**FIGURE 4:**
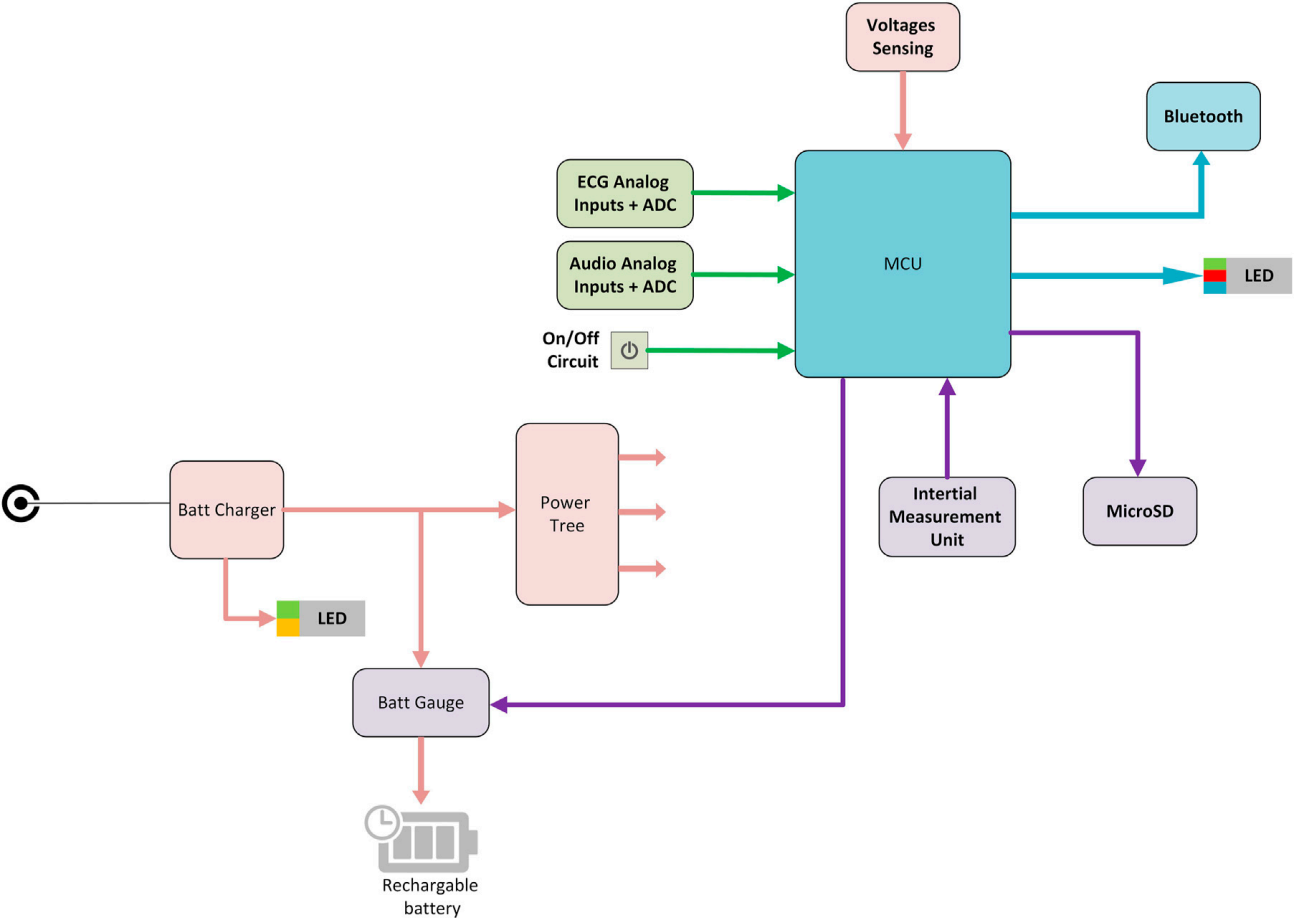
INVU electronic module block diagram. MCU–microcontroller unit; ADC–analog-to-digital converter.

The electronic module is the part of the system that acquires the physiological signals and transmits them to the mobile device. The general block diagram of the INVU electronic module is illustrated in Figure 4. The INVU wearable belt includes eight biopotential sensors (seven sensing sensors and a reference sensor) and four acoustic sensors. All sensors are connected to a 32-bit microcontroller running embedded software for data encapsulation. Physiological signals are acquired using multi-channel, 24bit, Sigma-Delta ADC, with input reference noise of 1uV RMS (at0–70 Hz) and an RTI noise of less than 1.5uVpp. The theoretical LSB of the ADC is less than 100 nV (calculated as the dynamic range divided by the number of bits). However, due to the noise levels, the effective LSB is 1.2uV. This value was experimentally obtained by grounding all input terminals and placing the electronics inside a Faraday cage. Higher LSB values are expected (up to 1.5uV) once the sensors are connected to the electronic circuit. All analog signals are sampled at 1 kSPS followed by applying a digital low-pass antialiasing filter (120 Hz cut-off) to the sampled signals. The signals are then downsampled by a rate of 1:4, resulting in a sampling rate of 250 SPS. A motion module is used in the system to detect maternal physical movement via an onboard inertial measurement unit (IMU). The motion module includes a three-axis accelerometer and a three-axis gyroscope and a built-in ADC which samples all motion signals at 50 SPS. All physiological and motion signals are encapsulated into data packets and transmitted via Bluetooth to the mobile device. The recorded motion signals are processed as described in Mhajna et al. (2020) to extract the maternal activity level over time. Additional hardware data are collected to provide battery status and general hardware functionality status.

### 2.3 Uterine Activity Algorithm

The CaBUM algorithm performs signal processing to identify maternal and fetal cardiac signals and uterine contractions by fusing the information gathered independently from the ECG and PCG sensors. Uterine contractions lead to conformational changes in the tissue through which the maternal signals travel, resulting in a signal modulation that correlates with the mechanical effect of the contraction and can be detected by the algorithm (Mhajna, 2019).

**FIGURE 5:**
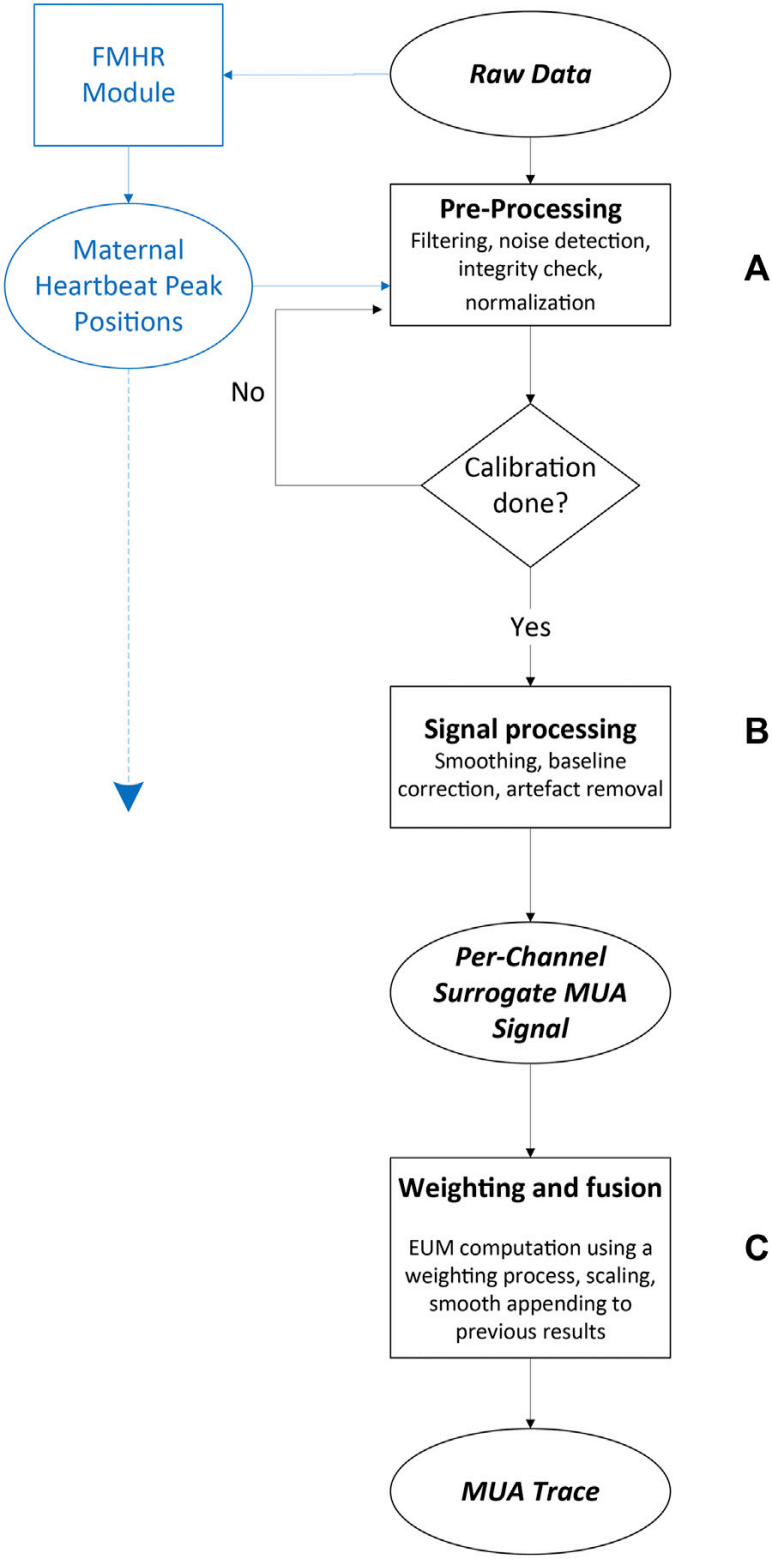
Maternal uterine contraction algorithm (black) and fetal and maternal heart rate algorithm (blue) components of the wireless, remote prenatal monitor. Details of the heart rate algorithm have been presented previously (Mhajna et al., 2020).

The algorithm for extracting UA from the ECG and PCG signals involved three main stages (Figure 5; a more detailed version of this diagram can be found in Supplementary Figure S2): 1. Data pre-processing, performed on both ECG and PCG signals. This stage included filtering, noise detection, integrity check, and normalization (Figure 5A); 2. Per-channel surrogate-UA preparation from heartbeat peaks (QRS complexes for ECG signal and sound-based peaks from the S1–S2 heart sounds of the PCG signal) and processing of these data: cleaning, scaling, correcting for baseline changes and abrupt changes, smoothing, contraction identification, and signal enhancement (Figure 5B); and 3. A channel weighting process and fusion of the per-channel surrogate UA into one finalized UA trace (Figure 5C). The first stage was performed on every 1-min section of recorded data, immediately during data collection, whereas the other two steps were accessed for the first time only after 10 min of recording, which were held for calibration before the UA trace was shown to the user for the first time. This allowed for a robust initial contraction detection, considering the low occurrence rate of uterine contractions. From that point onward, all algorithmic steps were performed on every newly acquired 1-min section of recorded data. These three steps are described in detail below.

#### 2.3.1 Data Pre-processing

**FIGURE 6:**
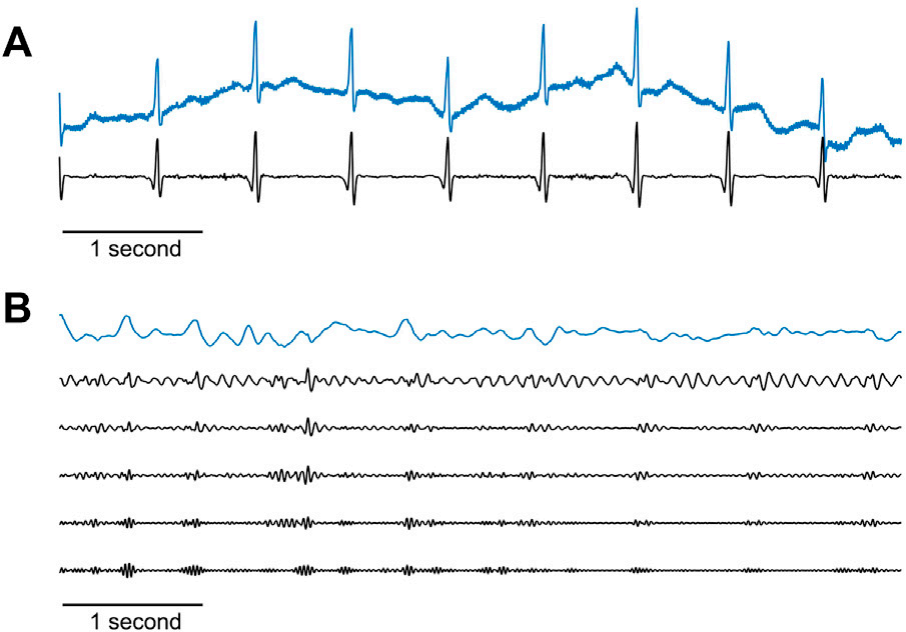
Raw maternal signals recorded by the sensors and their pre-processed versions. **(A)** Example of a 6-s raw biopotential signal (blue line) and the result after applying the filtration stage as described in the text (black line). **(B)** Example of a 6-s raw acoustic signal (blue line), and after passing both a 50 Hz cut-off frequency low-pass IIR filter, and an IIR high-pass filter with the following five cut-off frequencies (shown from top to bottom in the figure): 10, 15, 20, 25, and 30 Hz. For better visualization, these five filtered traces are scaled to the dynamic range of the frequencies in each trace.

The biopotential signals were filtered with a DC-blocking filter which subtracted the signal average, followed by an IIR notch filter to remove power-line noise, with a stopband of 0.5 Hz above and below the power-line frequency. An additional high-pass-like inverse, moving average filter was used to eliminate low-frequency noises. The duration of the filter was set to 201 milliseconds. The filter was applied by calculating a sliding average of the original signal, which extracted the low-frequency components of the signal using a convolution with a hamming window, and subtracting this average from the original signal, leaving the higher frequency signals. An example of a raw and filtered biopotential signal is shown in Figure 6A.

The PCG signals were passed through a low-pass IIR filter with a 50 Hz cut-off frequency. Next, five distinct time series were created from each acoustic signal by submitting each channel to IIR high-pass filters with the following five cut-off frequencies: 10, 15, 20, 25, and 30 Hz. This resulted in five sets of acoustic signals that differed in their spectral content, originating from the same recording. The goal of this signal replication was to increase uterine contraction detectability by broadening the search for maternal PCG signals in data with more diverse characteristics, ultimately improving the weighting process and ensuring the selection of the best surrogate-UA activity hereafter. Figure 6B shows an example of raw and filtered acoustic signals.

Next, an integrity check was performed, in which sensor contact problems were detected by using a trained support vector machine classifier that used as features 1) high root mean square (RMS) of the raw signals, 2) low signal-to-noise ratio (SNR) of the pre-processed signals, and 3) low heartbeat peak energy relative to background power in the pre-processed signals. Importantly, all these processing stages were performed on both biopotential and PCG signals.

**FIGURE 7:**
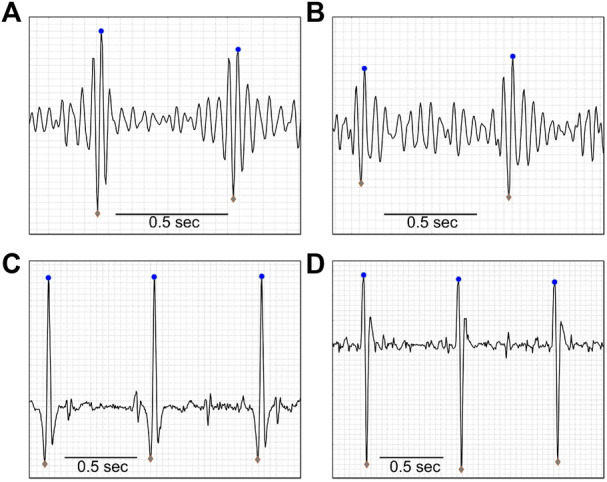
Heartbeat detection. Upward (blue dots) and downward (gray diamonds) pointing maternal heartbeat peaks, as detected by the algorithm, of a 19-year-old pregnant woman, at 38 weeks, with BMI = 36.7 kg/m^2^. Panes A and B show examples of acoustic data, and panes C and D show examples of biopotential data. Data were extracted after the pre-processing stage of the algorithm was completed.

Next, the heartbeat peaks from the current 1-min interval were processed on both biopotential and acoustic signals. Initial peak positions and values were received as inputs from prior algorithm modules and went through a refinement process to update their precise position and amplitudes, given the filters applied to the channel data as described above (Mhajna et al., 2020). Heartbeat peak data were separated to two series of time stamps and amplitude values: In the “upwards series,” an iterative process took a segment of data around each R-peak location detected previously, re-mapped the QRS complex, and extracted the accurate R-peak position and value as the extremum point in the segment. In the “downwards series,” the signal was inverted in order to detect R peaks in leads where the latter might be pointing downwards. As both upward and downward peaks were marked, Q, R, and S waves were detected for each QRS complex in the biopotential signals. Additionally, peaks in the acoustic channels were based on either the “lub” (S1) or the “dub” (S2) sounds of the heart (Gupta et al., 2007; Deng and Bentley, 2012; Gomes et al., 2013). We will therefore use the non-specific modal term “heartbeat peaks.” Examples of heartbeat peak detection from different biopotential and acoustic signals are shown in Figure 7.

A routine for fixing noise-corrupted regions operated on the signals, along with the maternal motion analysis output from previous algorithm modules (Mhajna et al., 2020). The result of this process could either be the rejection of a corrupt segment, or the replacement of noisy heartbeat peaks by values based on the median of the channel’s heartbeat peaks. A supplementary noise detection routine was performed at this stage. In this process, the algorithm correlated each heartbeat complex (QRS complex or S1–S2 [phonocardiogram sounds]) with an averaged heartbeat template and marked sub-threshold heartbeat windows as noisy ones. If no noisy instances were detected, the signal-to-noise ratio (SNR) was inspected for each heartbeat segment for further detection of noisy instances. The SNR was calculated for each heartbeat as the root mean square (RMS) of the QRS complex divided by the mean RMS between complexes. Channels containing low SNR segments that lasted more than a predefined threshold were removed entirely from further processing. Otherwise, low SNR segments were rejected specifically (samples were zeroed).

#### 2.3.2 UA-Surrogate Preparation

**FIGURE 8:**
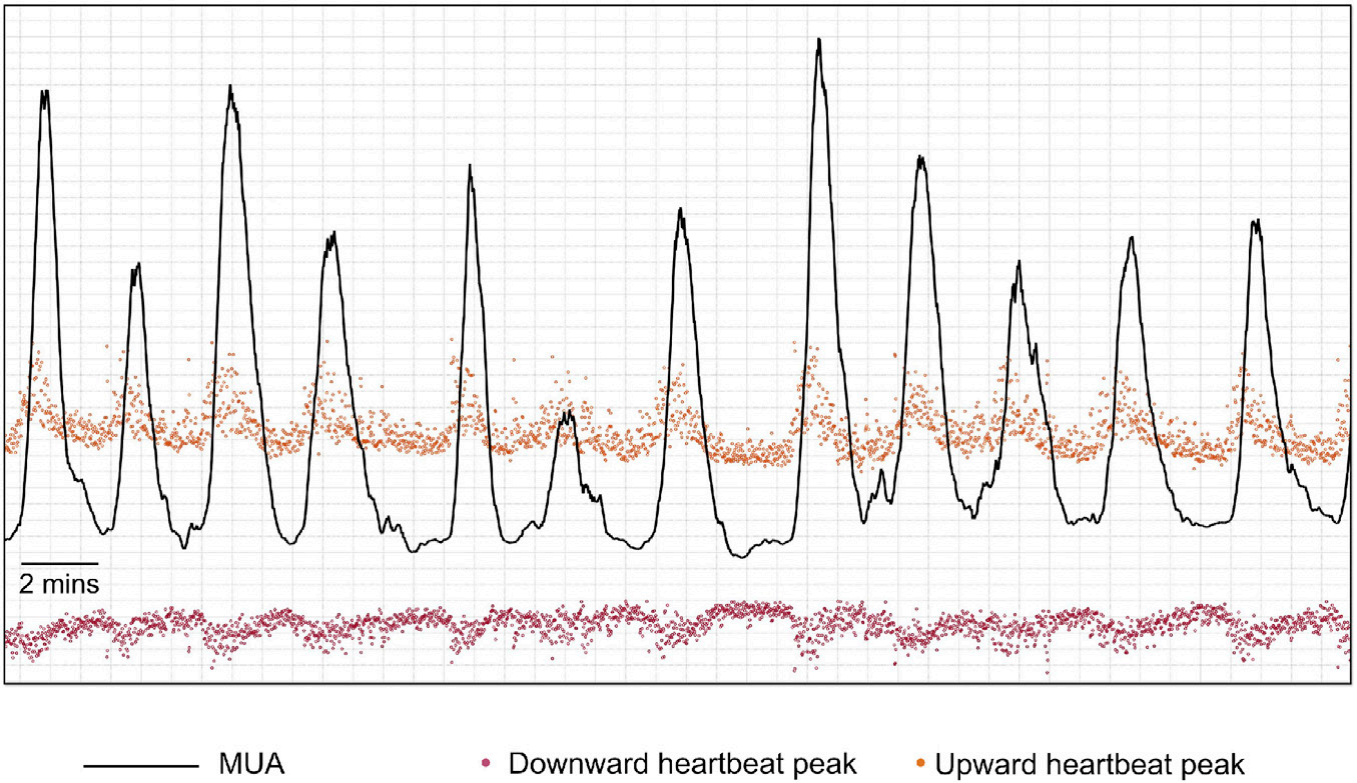
Example of an initial maternal uterine activity trace (MUA–black line) that is produced by a simple addition of the interpolated upward-pointing peaks’ time series (orange points after interpolation) and the absolute values of the interpolated downward-pointing peaks’ time series (red points after interpolation).

At this stage, the algorithm extracted and processed the envelope amplitude of the heartbeat series obtained above, which would constitute a surrogate measure for UA. Since the heartbeat peaks were discrete points in time, to process the peak data as a continuous time series, a cubic spline interpolation was performed on each channel’s upward peak and downward peak values to produce a continuous time series of peak amplitude modulation with a constant sampling rate of four samples per second. Then, for each channel, an initial surrogate-UA trace was produced by a simple addition of the interpolated time series (Figure 8). This heartbeat peak modulation signal was then smoothed using a moving RMS filter with a duration of 101 samples that was implemented as a sliding window filter that calculated the RMS of the samples inside the window.

The signals were then enhanced to further improve the clarity of the UA. First, the signal was adaptively shifted above 0 to include only positive values using a centered-moving minimum window that computed local minima along the signal and removed them point-wise. Channel data were then raised to the power of 2 to enhance local contraction activity. This non-linear transformation guaranteed a stretch of the transient contractions, which had a near-Gaussian shape, while keeping baseline activity between contractions low, without affecting the contraction time and duration.

Before creating a final UA trace by weighting and fusing the channel data, a preliminary contraction identification was performed in each of the channels by the automatic contraction identifier, and properties were extracted from these contractions for other uses. For each contraction, three confidence measures were calculated: 1. Relative energy of the contraction (area under curve). 2. Peak to range, calculated as the average of the upper third of a contraction divided by the value range of a contraction. 3. Contraction value range relative to the range of the non-contractile signal. These three measures of confidence were compared against pre-defined thresholds and were used to eliminate outlier contractions. Moreover, two scores were extracted for each contraction: 1. The difference between the UA before and after a contraction, divided by contraction peak amplitude; and 2. Normalized prominence, calculated as the difference between contraction peak amplitude and the mean UA before and after contraction, divided by the peak amplitude. These scores were used for setting up the initial weights in the next algorithmic stage.

#### 2.3.3 Channel Weighting and Fusion

The continuous envelope of the heartbeat amplitude obtained for each channel was an indirect surrogate measure for UA activity. To obtain a single final high-quality UA result, these traces were enhanced and averaged according to a set of weights based on channel quality criteria. A multi-start-point gradient descent (GD) scheme was utilized to obtain the optimal channel weights. Each start point corresponded to an initial set of weights. In each set, a subgroup of channels was selected to be weighted, while all the other channel weights were zeroed. The subsets that were selected as starting points were as follows: 1. The *biopotential subset* that consisted of biophysical channels only; 2. The *acoustic subset* that consisted of acoustic channels only; 3. The *combined subset* that consisted of all biopotential and acoustic channels; and 4. The *contractions-based subset* that used the previously detected contractions for each channel to cluster the channels into three groups using a K-Means clustering algorithm. The input features used were kurtosis, energy, skewness, rise and fall times, and duration of the contractions. The best cluster, taken as the one with the largest number of maximal values across features, was selected as the contractions-based subset.

For each of the checked sets of weights, the process of weight construction started with two series of initial weights:

(1) *Channel voting and contraction quality.* Here, the working channels (with weights that are not zero) voted, per sample, on other channels; the possible voting options for each sample were whether a contraction exists for this sample or not. Then, for each channel, all votes were counted and divided by the total number of voting channels. On top of that, the average reliability of contractions for each channel was calculated. The reliability of a contraction is defined as the relative contraction power (area under curve) compared to the overall power in the signal for a specific channel. The first initial weight was then defined as the average of these two measures. Later on, for processing frames beyond the first 10 min of recording, damping was applied to mitigate abrupt weight changes between processing frames, using:

$$w_{0}^{1}=0.6*w_{previous}+0.4*w_{current}.$$

(2) *All equal,* where all channels were given the same initial weight that equals:

w02= 1N ,

Where N was the number of included channels.

These two weight vectors were then independently optimized by a GD optimization function which adjusted the weights by iteratively minimizing a cost function. The weights vector, w , is updated using the following equation:

$$w_{n+1}=\left|w_{n}-\gamma_{n}\cdot J\left(w_{n}\right)\right|.$$

Here, $\gamma_{n}$ denoted the step size in the GD optimization scheme. The step size was decreased following a sigmoidal function that was optimized to increase the speed and accuracy of the GD scheme; $J\left(w_{n}\right)$ was the Jacobian matrix at the $n^{th}$ step. When updating the weights, the absolute value was taken to ensure that the weights did not become negative.

The cost function that was optimized is defined as:

$$C\left(w_{n}\right)=1+\frac{1}{2}\left(\frac{E_{n}^{cont}}{E_{n}^{tot}}+\frac{A_{n}^{cont}}{R_{n}}\;\right)\;.$$

At the $n^{th}$ step, a temporary UA signal was calculated using the current $w_{n}$ , by multiplying this weights vector with the signal’s matrix (weighted average). The overall energy of this signal was denoted as $E_{n}^{tot}$ . After performing contraction detection on this signal, the overall energy of the contractions $E_{n}^{cont}$ , the average amplitude of these contractions $A_{n}^{cont}$ , and the average range of these contractions $R_{n}$ were then calculated.

After optimization, the two weight vectors competed against each other, and the set of weights selected to “represent” the channel subset then competed with the weights of the other channel subsets toward a selection of the final weighting vector. Several numerical measures were calculated in the selection process. These measures included: 1) SNR of the resulting UA trace, 2) the values of the cost function for the weight vector, 3) the contraction confidence measures, and 4) a *difference index* defined as $\text{Difference Index}=1-max\left\{0,\;\rho \left(S_{prev}\;,\;S_{current}\right)\right\}$ . ρ was the Spearman correlation coefficient between the previously extracted UA signal from previous processing frames, if available, and the current UA signal.

**FIGURE 9:**
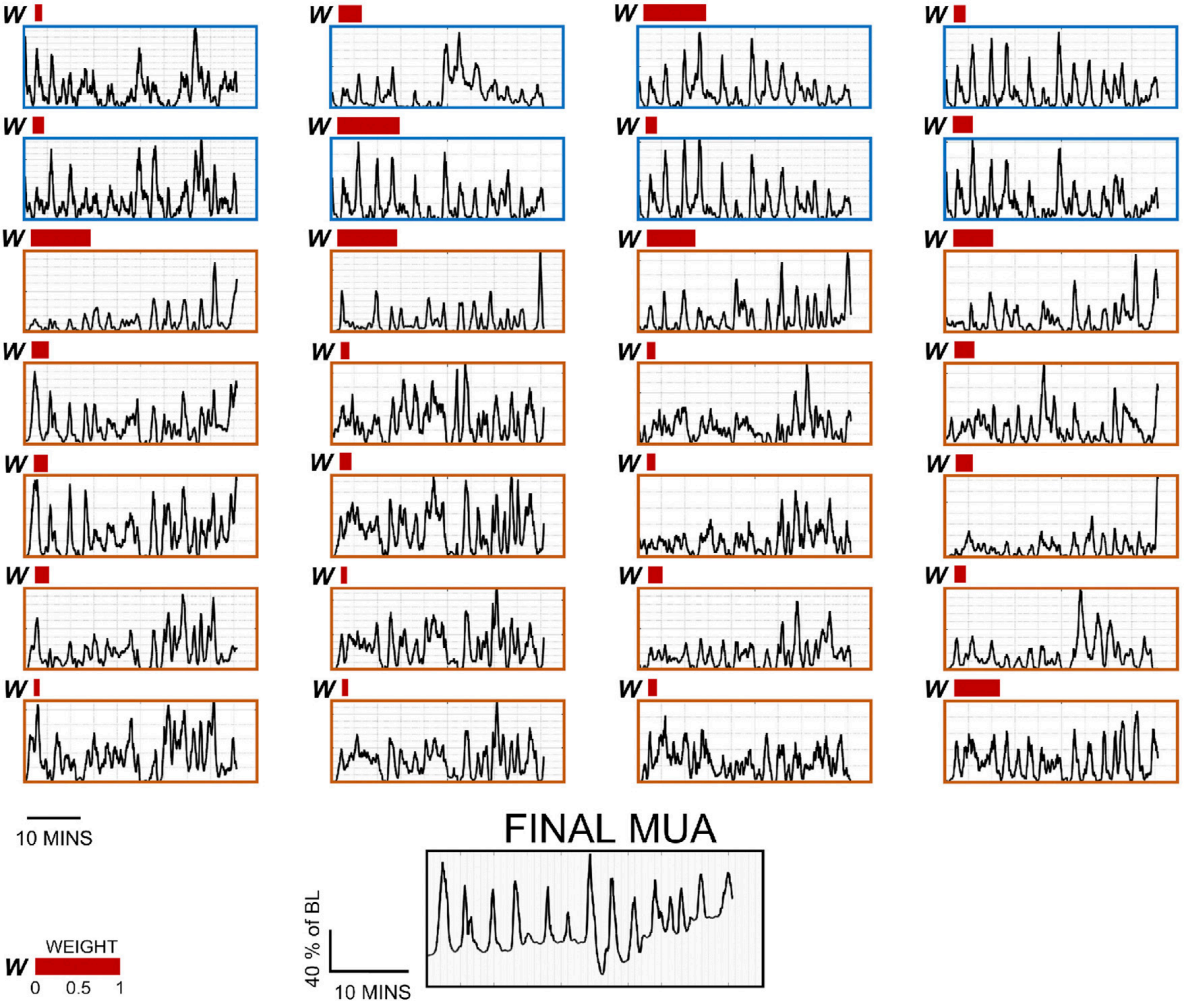
Per-channel surrogate MUA traces and weights for 28 channels, and final MUA activity from a representative subject. The first eight channels (surrounded by a blue frame) are biopotential channels, and the rest (surrounded by an orange frame) are acoustic channels under the different pre-processing parameters described in this study. Note that each 1-min data segment composing these data has their own weight distribution. The weights represented here (red bars) are taken from the last recording segment. Channels with no bars were given the weight of 0.

After selection of the final weighting vector for each set, two further steps were performed to improve the selection process before the algorithm selected the best set. The first step consisted of enhancing data in channels with marked contractions. For each channel, a measure of similarity with the weighted averaged signal was computed. For this, three metrics were examined: 1. Any correlation coefficient between the weighted average signal and the individual channels that exceeded a threshold; 2. The first parameter of the first-degree polynomial fit between the channel data and the weighted average; and 3. The estimate error (delta) of the fit. These three metrics were examined against predefined fixed thresholds, and the weights associated with any supra-threshold channels were retained. The rest of the weights were zeroed. Remaining weights were then scaled to sum up to 1. Then, an additional iteration of GD optimization was run on the weights resulting from the previous steps. The second step considered weights from previous processing frames if they existed. The current processing frame was assigned a contribution weight ( CW ) from 0 to 1, and the previous weights were assigned the complementary contribution weight ( 1−CW ). As more previous segments existed, the current segment would be assigned a lower CW (i.e., each additional recording segment added a 1/segment-number bit of information). Then, weights were adjusted to balance between current and previous frames according to this weighing method. The final channel set was selected using the same metrics used for the selection of weights within each subset and performing a pairwise comparison of each successive sets. An example for constructing the final UA segment from the chosen weights of the chosen channels set is shown in Figure 9. A demonstration of the evolution of the weights of two representative channels can be seen in Supplementary Figure S3.

Finally, for processing frames beyond the first 10 min, the algorithm performed a stitching process in which a new UA segment was appended to the previous UA segment. An adaptive baseline correction technique operated on the entire signal to compute the optimal baseline correction factor but was applied to the new segment only. As the last step, the current UA trace was shifted to the correct point where it was appended to the end of the previous part of the trace.

### 2.4 Experimental Verification

#### 2.4.1 Recording Protocol

The measurements used in this work were taken from two separate prospective, comparative, open-label, multi-center studies (Mhajna et al., 2020) (Schwartz et al., 2022). The studies were conducted in accordance with the principles set forth in the Declaration of Helsinki and in compliance with ICH-GCP standards. For both studies, all patients provided written informed consent to participate.

**FIGURE 10:**
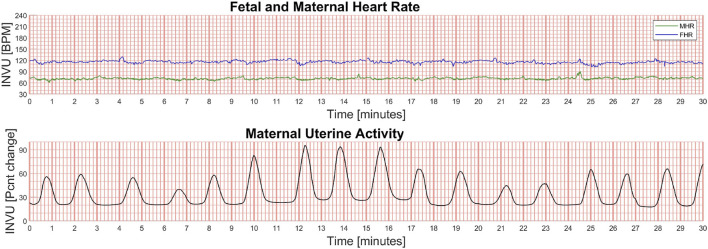
Example of the output of the INVU system. The fetal and maternal heart rates (in beats per minute [BPM]) are shown in the upper plot (green line is the MHR and blue line is the FHR), and the maternal uterine activity (MUA) is shown in the lower plot. The MUA trace is unitless and displays the % change in activity from baseline.

1. Study 1 (termed *“Intrapartum study”*) assessed the agreement between INVU and IUPC (two-way setup) plus TOCO, if applicable (three-way setup) (NCT03889405). Females between the ages of 18 and 50 years were eligible to participate in this study after they met all inclusion criteria: a singleton pregnancy with gestational age ≥32 weeks, being in the first stage of labor and having an IUPC in place. The local Institutional Review Board at each study site approved the protocol (University of Arkansas, protocol 229056, approved 3/25/2019; University of Pennsylvania, protocol 832522, approved 3/6/2019). In the two-way sessions of the intrapartum study, IUPC and INVU were recorded simultaneously. In sessions where TOCO was also present, the CTG transducer was placed after the INVU belt was positioned on the abdomen, and uterine activity was recorded simultaneously from all three devices. UA was measured continuously for a duration of 30–60 min.
2. Study 2 (termed “Antepartum study”) compared simultaneous recordings of INVU andTOCO in pregnant women aged 18–50 years, with singleton pregnancies and a gestational ageof ≥32 weeks (NCT03504189). The local Institutional Review Board at each study site approved the protocol (Hadassah-Hebrew University Medical Center: EC # HMO-0116-17, MoH# 20174697; approved 1/17/2018; Heidelberg University: CIV-17-05-019406; approved 3/26/2018; University of Pennsylvania IRB: PROTOCOL#: 828202; approved 10/26/2017; EVMS: Chesapeake IRB Pro00022598; approved 11/10/2017). In this study, the authorized study personnel applied both INVU and TOCO transducers, and a 30-min session was initiated recording from both systems simultaneously. An example of the outputs of INVU are shown in Figure 10 which depicts a recording of the FHR and MHR, together with a UA trace.

#### 2.4.2 Evaluating Results

For the antepartum study, TOCO traces and contractions were used as the reference dataset, while for the intrapartum study, IUPC traces and contractions served as a reference.

An automatic contraction identification algorithm (described in Supplementary Section A) was used for detecting contractions on the recorded INVU and TOCO traces. The detection process was run in the same manner for all three device types. To compare the contractions detected in the intrapartum study, each contraction identified from the INVU (or TOCO) trace was compared and matched to a single corresponding contraction in the IUPC trace. Additionally, it was confirmed that each contraction in the IUPC trace was matched to a single contraction in the other methods under investigation. To define a contraction as a match (i.e., true positive), the temporal overlap of the two contractions under investigation needed to be either at least 30 s or 50% of the total contraction duration, whichever was shorter. In case multiple contractions in the method under investigation matched a single contraction in the IUPC trace, the contraction with the maximal temporal overlap was taken as the matched contraction. In the antepartum study, TOCO was defined as the reference device, and each contraction identified from the INVU trace was compared and matched to a single corresponding contraction in the TOCO trace, in the same way as above. As the goal of this analysis was to demonstrate the performance of the CaBUM algorithm in evaluating UA, only sessions where the automatic detector identified at least one significant contraction (amplitude ≥15 mmHg above the baseline) on the TOCO trace were included in the analysis.

As measures of *sensitivity*, we determined the positive percent agreement (PPA) for both external devices (INVU and TOCO) relative to the comparator(s). The PPA indicated the percentage of true contractions detected by the external device, from the total number of contractions detected by the reference method. Since each patient could have a different number of contractions, the PPA was calculated for each patient separately, and then a weighted average was performed, with weights calculated as the number of contractions in the corresponding IUPC trace divided by the total number of IUPC contractions for all relevant patients. The standard deviation (SD) was calculated in a similar manner. To assess the *false-positive rate*, we calculated the percentage of falsely identified contractions by the external device (false discovery rate [FDR]) from the total number of contractions detected by this device. Differences in the sensitivities of INVU and TOCO among all patients were evaluated using the non-parametric Mann–Whitney *U* test. A two-tailed Pearson correlation analysis was performed to test the correspondence between the waveforms of the INVU and TOCO traces to the IUPC traces. The correlation was calculated separately for each waveform trace, and the result coefficients were then averaged.

## 3 Results

To objectively evaluate the diagnostic value of INVU, we evaluated its performance in comparison to the standard-of-care TOCO and the gold-standard IUPC. Since IUPC can only be used when the membranes are ruptured, this study required an intrapartum cohort. Additionally, as INVU is indicated for use on women who are ≥32 gestational weeks, it was also tested in an antepartum population.

### 3.1 Intrapartum Dataset

#### 3.1.1 Dataset

**FIGURE 11:**
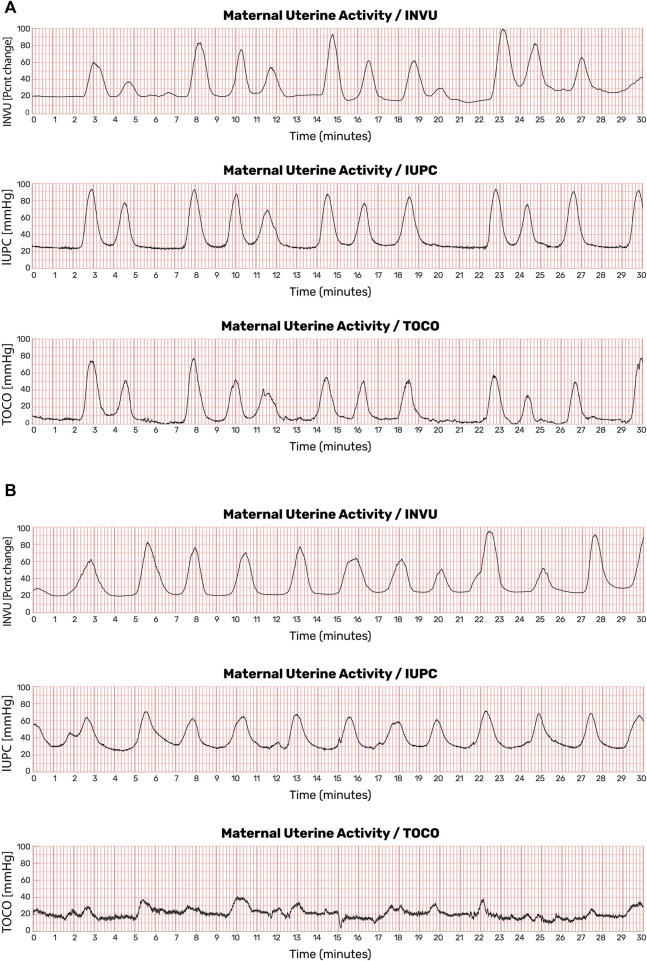
Uterine contraction monitoring sessions showing recordings from IUPC, TOCO, and INVU. **(A)** Both INVU and TOCO recordings followed the IUPC recording closely. **(B)** In some monitoring sessions, the TOCO tracing, which is more sensitive to positioning, motion, and placement, failed to show some of the IUPC contractions that were correctly identified by the INVU (this figure was published in N. Schwartz et al., “Novel Uterine Contraction Monitoring to Enable Remote, Self-administered Non-stress Testing,” Am. J. Obstet. Gynecol., 2021).

Demographic characteristics of the subjects who participated in the intrapartum study are listed in Supplementary Table S1. Overall, for the total of 41 subjects, the average (±SD) gestational age was 38.8 ± 1.5 weeks, the average maternal age was 26.7 ± 5.2 years, and the average pre-pregnancy BMI was 29.6 ± 7.7 kg/m^2^. The two-way subset included a total of 31 subjects, and the three-way subset included a total of 10 subjects. The demographic characteristics of the patients were similar across the two setups. Figure 11A shows the results of a 30-min session with one participant. The automatic contraction identification algorithm was used to detect contractions on all three methods. The contractions detected on the external methods (INVU and TOCO) were then compared with the contractions detected on the IUPC trace. Figure 11B shows traces from another subject in which INVU closely followed IUPC, while TOCO missed most of the contractions.

#### 3.1.2 Experiment and Results

A total of 1,412 recording minutes of uterine activity data were collected: 1,046 min in the two-way, and 366 min in the three-way setups. A total of 557 contractions were detected on the IUPC traces, with 500 of them detected on INVU traces. Thus, the overall PPA was 89.8 ± 14.5% (mean ± SD). Moreover, 47 surplus contractions were identified on INVU traces and were not detected on IUPC, yielding an FDR of 8.6 ± 12.1%. Analyzing the three-way sessions revealed a significantly higher percentage of true contractions detected on the INVU traces: of the 135 contractions detected on IUPC, 126 were detected on INVU traces yielding a PPA of 93.3 ± 12.6%. This result is significantly greater than for TOCO, where only 52 contractions were detected of the 135 detected on the IUPC traces, resulting in PPA of 38.5 ± 45.5% (*p*-value = 0.0054). For the FDR in the three-way setup, of the total 138 contractions detected on INVU, 12 were not detected on IUPC resulting in an FDR of 9.1 ± 11.8%, while for TOCO, of the 53 total detected contractions, only one contraction was not detected on IUPC, resulting in FDR of 1.9% (Table 1).

**TABLE 1:** PPA, FDR (both values are mean ± SD), and correlation to IUPC for TOCO and INVU devices in the intrapartum study.

Group	INVU			TOCO		
	PPA (%)	FDR (%)	R (correlation to IUPC)	PPA (%)	FDR (%)	R (correlation to IUPC)
All sessions	89.8 ± 14.5	8.6 ± 12.1	0.79 (95% CI 0.73-0.84)	NA	NA	0.35 (95% CI 0.13-0.57)
Two-way only (n = 31)	88.6 ± 15.2	8.4 ± 12.4		NA	NA	
Three-way only (n = 10)	93.3 ± 12.6	9.1 ± 11.8		38.5 ± 45.5	1.9 ± NA	

Finally, to quantify the similarity of the waveforms in the three-way setup, the correlation of INVU and TOCO traces with the corresponding IUPC traces was calculated and found to be 0.76 (95% CI 0.65–0.87) for INVU and 0.35 (95% CI 0.13–0.57) for TOCO, a significantly higher value for INVU compared to TOCO (-value <0.005).

As a secondary analysis, we assessed the influence of obesity on the performance of INVU and TOCO. The performance of TOCO significantly decreased in the case of higher BMI. INVU on the other hand had a considerably better performance (higher sensitivity and lower FDR) for patients with higher BMI (Table 2).

**TABLE 2:** PPA and FDR (mean ± SD) by BMI prior to pregnancy in the intrapartum study, for total INVU data, and the mean for both devices (INVU and TOCO) in the three-way study. Total INVU data.

BMI group	PPA (%) mean ± SD	FDR (%) mean ± SD
Normal (BMI<25, n = 14)	85.9 ± 17.9	11.7 ± 15.0
Overweight (25 ≤ BMI<30, n = 9)	95.7 ± 05.8	08.9 ± 12.6
Obese (BMI≥30, n = 18)	89.8 ± 14.2	06.1 ± 09.3

BMI group	INVU		TOCO	
	PPA (%)	FDR (%)	PPA (%)	FDR (%)
Normal (BMI<25, n = 4)	90.4	7.8	53.8	0.0
Overweight (25 ≤ BMI<30, n = 2)	96.0	17.2	20.0	0.0
Obese (BMI≥30, n = 4)	94.8	5.2	32.8	5.0

### 3.2 Antepartum Dataset

#### 3.2.1 Dataset

A total of 147 recording sessions from 147 different subjects were collected. Of these sessions, 10.9% (16 of 147) were detected to have at least one significant contraction on the TOCO trace by the automatic contraction identification algorithm and were included in the analysis. Demographic characteristics of these 16 subjects in the antepartum study are listed in Supplementary Table S2. The average gestational age of the subjects was 37.2 ± 2.2 weeks, comparable to that of the intrapartum data. The average age was 33.18 ± 7.06 years, and the average pre-pregnancy BMI was 23.3 ± 3.5 kg/m2, both slightly different for the antepartum compared with that of the intrapartum subjects, however, still comparable.

#### 3.2.2 Experiment and Results

**FIGURE 12:**
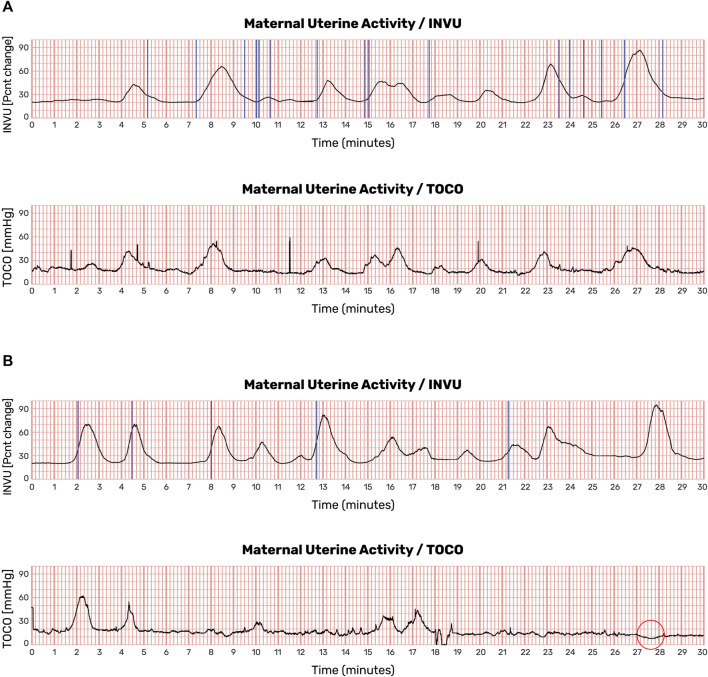
UA monitoring sessions showing recordings from TOCO and INVU during antepartum stage. Contractions detected by INVU and TOCO displayed along with the mother’s reports of contractions she felt. **(A)** INVU trace closely follows TOCO trace. The blue vertical lines represent contractions felt by the mother. **(B)** INVU trace shows all contractions presented on both TOCO trace and the mother's perceptions and additional contractions that are neither identifiable by TOCO nor perceived by the mother (false-positives). The red circle denotes a negative deflection at the TOCO trace that appeared concurrently with a contraction identified by INVU.

Antepartum UA data were collected for a total of 456 min. Examples of such UA are shown in Figure 12. In this figure, the contractions detected by INVU and TOCO are displayed along with the mother’s reports of noticeable contractions. In Figure 12A, the INVU trace closely followed the TOCO trace and performed, in terms of sensitivity, at least as good as TOCO. In other cases, the INVU trace captured all contractions present on both the TOCO trace and the mother's perception, and additional contractions that were neither identifiable by TOCO nor perceivable by the mother (Figure 12B). An interesting phenomenon in this example is the negative deflection appearing on TOCO trace, concurrently with the last contraction in INVU’s trace.

Since IUPC was not available in the antepartum stage, there was no comparable gold standard for evaluating which of the two external methods had performed better; hence, TOCO was defined as the reference device, and the sensitivity of INVU for detecting TOCO-defined contractions was examined. A direct comparison of INVU to TOCO in the antepartum study showed that of the 95 contractions detected by TOCO, 89 of them were also detected on the compatible INVU traces, resulting in a PPA of 94 ± 8.7% (95% CI 89.7%–98.3%). Additionally, of the total 129 contractions detected on INVU, 40 were not detected on TOCO, resulting in an FDR of 31.1 ± 25.8% (95% CI 17.5%–42.7%).

## 4 Discussion

In this study, we have introduced a novel approach for monitoring UA. The proposed CaBUM algorithm is a promising new way of tracking uterine contractions. Our study was based on recordings of UA taken directly from human subjects from both intrapartum and antepartum stages and was intended to compare INVU’s detection of UA with that of the gold-standard IUPC and the prevailing standard of care, TOCO.

A central finding of this study is that the performance of the CaBUM algorithm for monitoring UA outperforms the performance of TOCO. INVU correctly identified 500 contractions of the 557 reference (IUPC) contractions detected by an automatic marking algorithm, yielding a sensitivity of 89.8%, while the sensitivity of TOCO was 38.5%. Results from blinded, human assessors reviewing the same dataset (Mhajna et al., 2021) comparing INVU to IUPC are also promising, showing a similar sensitivity of 87.7%. A high sensitivity of 94% was also observed for INVU in the *antepartum* study, with TOCO as reference. Importantly, the high “false” detection rate of INVU in the *antepartum study* (31.1%) is likely largely explained by the low sensitivity demonstrated for TOCO in the *intrapartum* study. As INVU was shown to have a significantly higher sensitivity than TOCO, it is expected to capture more IUPC-detected contractions compared to TOCO, resulting in a high FDR when directly comparing INVU to TOCO. Indeed, TOCO has been shown to be less accurate when compared with IUPC (Hayes-Gill et al., 2012; Euliano et al., 2013; Hadar et al., 2015; Nguyen et al., 2016; Vlemminx et al., 2017; Cohen and Hayes-Gill, 2014), since its sensitivity is hampered by obesity and maternal movements (Bakker et al., 2007; Vlemminx et al., 2018; Euliano et al., 2013; Euliano et al., 2007; Ray et al., 2008). The consistency in INVU’s sensitivity with different references in the two studies further strengthens the assumption that INVU performs similarly in both the intrapartum and antepartum settings. In this context, it should be noted that the selection of TOCO traces that demonstrated contractions in the *antepartum study* may impact the FDR results reported here. However, the lack of an accurate reference such as IUPC in the antepartum stage does not allow for the estimation of the true FDR of the INVU device, leaving the TOCO sessions with clear contractions as an efficient compromise.

The INVU system uses an innovative technology to extract ongoing UA, based on the sensitivity of the amplitude modulation of the cardiac signals to UA, such that a trace of contractions can be obtained by processing the maternal ECG and PCG signals. We hypothesize that when a contraction occurs, three distinct mechanisms modulate the ECG and PCG signals that are propagating through the body: 1) displacement of the heart; 2) changes in skin impedance, both electrical and acoustic; and 3) changes in the body media. Supportive evidence for this hypothesis can be found in the reported behavior of specific resistance parameters of the uterus, such as the medium stiffness, its permittivity, and permeability. These parameters are known to affect biopotential and acoustic signals and were shown to change in response to uterine contractions (Moslem et al., 2011; Alamedine et al., 2013; Gennisson et al., 2015; Liu et al., 2017), thus affirming the effect of contractions on the ECG and PCG signals.

The idea of measuring modulations in ECG to get a robust physiological signal was already successfully implemented for obtaining respiratory signals (Ruangsuwana et al., 2010; Al-Khalidi et al., 2011; Chan et al., 2013; Helfenbein et al., 2014). The respiration cycle consists of many simultaneous processes affecting the ECG: the heart rotates in multiple dimensions, the heart-to-electrode distances change during thoracic expansion, and changes in thoracic impedance occur as air fills spaces in the lungs. All these factors create a modulating effect on the ECG, from which the respiratory signal can be extracted. In addition, it is widely reported that the respiratory modulations of signals such as ECG and photoplethysmography (PPG) are manifested through several modulation mechanisms such as amplitude modulation (AM), baseline wandering (BW), and frequency modulation (FM), whose strengths and weights depend on the breathing patterns and measurement sites (Charlton et al., 2018; Liu et al., 2020). As in the CaBUM algorithm, here too, the focus on modulations of the ECG signal sets the ground for a highly accurate signal and strengthens robustness against noise. However, there are two main differences between the UA- and respiratory-related modulations of the ECG and PCG signals: speed and periodicity. Respiration is a fast signal while UA is a very slow signal. On average, for every three heartbeats, the respiration signal finishes a cycle, while a contraction signal could span over 120 heartbeats. Also, the respiration signal is semi-periodic while the UA and contractions signal is non-periodic or event driven, thus the main modulation of the UA signal is mainly AM. In some pregnant women, uterine activity may also be associated with periodic changes in MHR (FM mechanism) (Odendaal et al., 2018) (Ibrahim et al., 2015), however, the AM mechanism is the one most commonly observed.

Modulations in the abdominally recorded ECG signal might originate from sources other than uterine contractions. For example, change in the mother’s heart activity due to exercise (Plews et al., 2013; Shaffer and Ginsberg, 2017), stress (Kim et al., 2018), or other normal and abnormal physiological conditions (Baselli et al., 1987; Taralov et al., 2016; Agliari et al., 2020). Analyzing and verifying the exact source of these modulations is critical to determining the false-positive rate of the proposed method. An effective way to overcome this issue is to trace the contractions in several physical modalities. The INVU sensor band records signals from two different types of sensors: biopotential and acoustic sensors. In addition to serving as a mechanism for increasing detectability, this dual modality also serves as an internal validation mechanism, verifying that the R-wave modulations originate from uterine contractions and not from changes in the mother’s heart activity as mentioned above. One of the major sources of false positives in external monitoring devices is maternal movements. To overcome it, the CaBUM algorithm uses the maternal motion analysis output from previous algorithm modules (Mhajna et al., 2020) as a filter for contractions-like motion artifacts.

In the last few decades, several techniques have been adopted for uterine activity monitoring during pregnancy and labor. The IUPC is currently the gold standard for measuring changes in the amniotic fluid pressure induced by uterine contractions. Unfortunately, this invasive method requires ruptured membranes and can only be performed by an experienced obstetrician. The most widespread alternative is TOCO, which non-invasively measures changes in the abdominal shape induced by uterine contractions. However, the accuracy of TOCO is low and highly dependent on proper positioning on the maternal abdomen, and its sensitivity is adversely influenced by maternal obesity (Hayes-Gill et al., 2012; Euliano et al., 2013; Hadar et al., 2015; Nguyen et al., 2016; Vlemminx et al., 2017). EHG uses electrodes placed on the maternal abdomen to evaluate the myometrium activity by measurement of biopotentials underlying uterine contractions. EHG has recently become available as a non-invasive alternative, but its signal quality depends on good skin preparation and correct position of the electrodes on the abdomen of the pregnant woman (Alberola-Rubio et al., 2013; Marchon et al., 2018; Rooijakkers et al., 2014; Tam and Webster, 1977).

**FIGURE 13:**
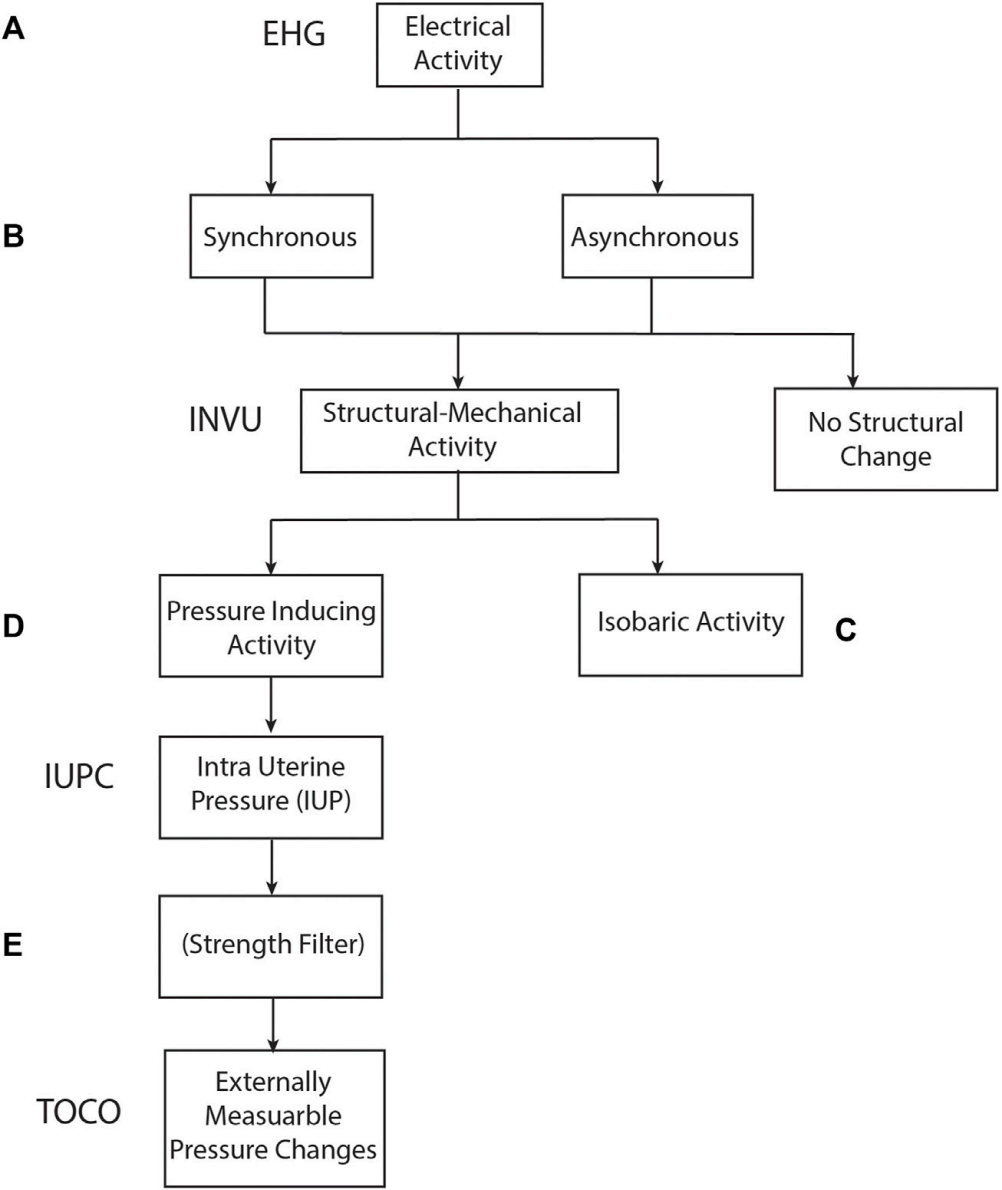
Relationship between physiological processes involved in uterine contractions and measurement methods used to identify contractions.

The abovementioned monitoring methods, together with the remote non-invasive INVU monitor, utilize different levels of the physiological contractile process for measuring the UA. Figure 13 sheds light on the stages involved in the physiological contractile mechanism and associates each stage with the device that extracts UA according to the information generated from that stage: The cascade of events that leads to a contraction originates with electrical activity in the myometrium (Figure 13A). This electrical activity may be captured by EHG-based methods which capture the overall significant electrical activity of the myometrium. Depending on the connectivity level of the myometrial myocytes, this electrical activity may consist of local asynchronous foci, or alternatively, the myocytes may behave as coupled oscillators and generate a more coordinated electrical activity (Figure 13B). In some cases, the cell activity of the smooth muscle remains focal and dissipates without creating structural changes to the myometrium, hence not identifiable by INVU. As the pregnancy progresses, several events such as increase in gap junctional surface area and their permeability combine to dramatically increase the connectivity of myometrial myocytes, making them more likely to concurrently depolarize and remain depolarized for longer (Hertelendy and Zakar, 2004). The stronger the connectivity between the myometrial myocytes, the larger the likelihood that they will behave as coupled oscillators and generate coordinated contractions leading to a structural change in the myometrium that may be identifiable by INVU. One of several scenarios may follow the myometrial electrical activity-induced mechanical changes: In some cases, the myometrial activation may lead to the generation of an isobaric contraction (Dobrin, 1973) that does not increase intrauterine pressure (Banney et al., 2015; Young and Barendse, 2014) (Figure 13C). However, in this case, a structural change in the myometrium does take place and as a result, alters the propagation of electrical and acoustic signals through the body tissue (Moslem et al., 2011; Alamedine et al., 2013; Gennisson et al., 2015; Liu et al., 2017). This change in propagation is ultimately reflected as a modulation of the ECG and PCG signals captured by INVU’s abdominal sensors. Nonetheless, as these structural changes in the uterus do not induce pressure changes, this activity would be reported as a false positive because it would not be detected by IUPC. When the contraction of the myometrium induces pressure variation of the amniotic fluid, this effect may be measured by IUPC device (Figure 13D). Finally, when the change in the intrauterine pressure is strong enough to induce changes to the maternal abdominal wall, these changes may be detected by an external strain-gauge transducer positioned on the maternal abdomen (Figure 13E). Understanding the mechanisms of action of the different UA monitoring devices, therefore, enriches our understanding of the physiological steps in the process of contraction generation, and may improve the computational models (Xu et al., 2022).

In the two-way setup of the *intrapartum study*, INVU exhibited an FDR of 8.4% (Table 1). As described above, it is possible that some of these surplus contractions detected by INVU may be derived from myometrial activation leading to the generation of an isobaric contraction that does not alter the intrauterine pressure, but still results in a structural change that can alter the propagation of electrical and acoustic signals through the tissue. Such a contraction can still be detected by INVU’s abdominal sensors; however, as these mechanical changes do not induce pressure changes, this activity would not be detected by IUPC or by TOCO and could therefore be reported as a false positive (Schwartz et al., 2022). A similar phenomenon is also relevant to explain the reported FDR in EHG-based devices (up to 21.4% (Vlemminx et al., 2017; Hadar et al., 2015; Cohen and Hayes-Gill, 2014)), which are triggered by the myometrial electrical signal, even if no significant muscle contraction ensues (Jacod et al., 2010).

Analyzing the intrapartum data also revealed a significant decrease in the performance of TOCO in overweight and obese subjects. It should be noted that due to the small sample size, clear conclusions cannot be drawn here, yet a trend is noticeable. These results replicated our previous finding (Schwartz et al., 2022), showing a significant reduction in TOCO’s positive agreement for obese group compared to normal weight group, whereas the positive agreement of INVU did not vary across different BMI groups.

EHG is a promising non-invasive technology which is currently being investigated to improve external uterine monitoring. This technique reveals a high sensitivity for contraction detection during term labor, similar to the sensitivity obtained by INVU. However, the CaBUM algorithm presented here offers several advantages over the utilization of EHG. First and foremost, INVU uses both biopotential and acoustic sensors, contrary to the unimodal data extracted by the EHG. The use of multi-modal data increases the accuracy and reliability of the extracted UA signal, and was also shown to improve FHR signal loss (Mhajna et al., 2020), especially in the 26th to 32nd weeks of gestation when the vernix caseosa masks the electrical fetal signals (Verdurmen et al., 2016). Moreover, INVU’s CaBUM algorithm is based on the ability to capture the maternal ECG and PCG signals, a relatively easy task, making it less prone to “false” contractions. The EHG, on the other hand, assumes low interference in the frequency domain of the EHG signal. This assumption is not always met, especially in a home environment.

EHG struggles to overcome the interference of electromyography (EMG) from other abdominal muscles, especially during the second stage of labor when the pregnant woman is actively pushing. It was reported previously that there is a decrease in the diagnostic value of EHG from the first to the second stage of labor (Vlemminx et al., 2017;Hayes-Gill et al., 2012). The CaBUM algorithm on the other hand is less prone to these interferences due to its ability to filter out motion-induced signal artifacts.

Many of the devices that utilize the EHG signal for monitoring UA also aim to capture the FHR from the fetal ECG signal using the same set of electrodes. The literature suggests that this could be a challenge (Rooijakkers et al., 2014) as there exists a tradeoff between the optimal sensor position for UA and FHR monitoring. Technically, to improve the SNR of the fetal ECG recording, the sensors need to be placed as far apart as possible to maximize the spatial filtering of electrical noises (Rooijakkers et al., 2014). On the contrary, to capture the EHG signal with sufficient quality, the electrical sensors should be positioned closer together (Alberola-Rubio et al., 2013; Gao et al., 2017). This conflict is mitigated in INVU by refraining from utilizing the EHG signal and rather focusing on improving the capturability of the fetal and maternal ECG signals, an essential element specifically for NST recording, in the antepartum stage where the FHR signal is less prominent.

There are a number of study limitations worth mentioning. A limitation of the *intrapartum* study is that participants had an IUPC in place for clinical reasons, associating them with a specific clinical group. The relieving presence of the IUPC might have caused the medical staff to reduce their efforts to carefully find the optimal location to position the TOCO transducer, therefore producing sub-optimal TOCO traces. This is evident when observing the decreased sensitivity of TOCO in our study compared to the literature (Hayes-Gill et al., 2012; Euliano et al., 2013; Hadar et al., 2015; Nguyen et al., 2016; Vlemminx et al., 2017). It is also possible that the placement of (either or both) TOCO and INVU devices was affected by the presence of the other external device. Such restriction may have also contributed to the low sensitivity of TOCO in this study. Furthermore, the inclusion of many participants undergoing labor induction with epidural analgesia is known to reduce patients’ restlessness, may lower the risk for mechanical artifacts, and could have improved both the sensitivity and the FDR of INVU. In the *antepartum* study, the main limitation was the inability to compare results to the IUPC gold standard.

The potential clinical applications of the proposed method for monitoring UA in pregnancy and labor are extensive. As INVU’s self-administered sensor band can operate remotely from the clinic or hospital, this platform could address several current limitations in pregnancy healthcare, such as remote monitoring during the early stages of labor, allowing the pregnant woman to remain at a supportive, relaxing, and homey environment while being monitored by the healthcare provider should any issue arise. Additionally, women with high-risk pregnancies, who require frequent fetal surveillance in the final months of pregnancy (Holness, 2018), could also reduce their travel and time burden with the use of the remote INVU platform. A further important implication relates to the useful and complementary diagnostic information that might be provided by the INVU device. On top of the physiological data extracted by INVU, a collection of rich measurements and indices calculated by the CaBUM algorithm is also available. Such features can be combined using a machine learning framework to gain a wireless remote classification of uterine behavior under different maternal conditions, similar to the effective predictions of maternal and fetal risks that are generated by the EHG method (Garcia-Casado et al., 2018; Asmi et al., 2019).

## 5 Conclusion

Uterine activity monitoring is an essential diagnostic tool during both antepartum and intrapartum periods. The current methods for monitoring UA need constant bedside presence, which increases the burden of work on healthcare providers. TOCO is affected by low accuracy and high dependency on proper positioning; IUPC is invasive and requires ruptured membranes; EHG is a promising method, however, it presents an interpretation challenge, given the interference of EMG from other abdominal muscles and the sensitivity to the electrodes’ position.

There is a pressing need for telehealth solutions in pregnancy monitoring, underscored by the current COVID-19 pandemic. This study has introduced a new method for measuring UA, based on uterine-induced bimodal modulations of the maternal ECG and PCG signals. This novel method has been demonstrated to show high sensitivity for detecting uterine contractions when compared with IUPC and exceeds that of the current standard-of-care-TOCO, staging it as a possible alternative to the current standard of care. Taken together with the existing remote FHR monitoring capabilities of INVU, the remote availability of the INVU non-invasive alternative for UA monitoring paves the way to a much-needed telehealth solution for pregnancy monitoring and has many promising possibilities in the field of diagnostic biomarkers based on its various calculated features.

## Data Availability

The raw data supporting the conclusion of this article will be made available by the authors, without undue reservation.
