# Supplementary material for: A Novel, Cardiac-Derived Algorithm for Uterine Activity Monitoring in a Wearable Remote Device
Source: Front Bioeng Biotechnol. 2022 Jul 19;10:933612. doi: 10.3389/fbioe.2022.933612 (PMC9343786; doi:10.3389/fbioe.2022.933612)
Supplement: Supplementary file 1 [file DataSheet1.pdf]

Supplementary Material

A Novel, Cardiac-Derived Algorithm for Uterine Activity Monitoring in a Wearable Remote Device

Muhammad Mhajna, Boaz Sadeh, Simcha Yagel, Cristoph Sonn, Nadav Schwartz, Steve Warsof, Leonardo Sandler, Yael Zahar and Amit Reches

A. Automatic contraction identification algorithm

This algorithmic module was used in both studies to detect contractions from all three UA measurements methods: intrauterine pressure catheter (IUPC), tocodynamometry (TOCO) and INVU wireless, remote prenatal monitor. The contraction identifier accepted three input signals: the original input trace, and two supplementary versions of the signal that were prepared ad-hoc for the detection process as follows: 1. Smoothed signal, obtained by convolving the first derivative of the original signal with a Hamming window and returning the cumulative sum of the result; and 2. Enhanced signal, obtained by computing the hyperbolic tangent of the z-score-normalized smoothed signal above. The result was a smoothed time series, where transient modulations in heartbeat peak amplitudes were readily manifested and detectable (**Supplementary Figure S1**). The contraction identifier implemented a peak detection algorithm that operated on the three signals. Outlier peaks were discerned via their prominence, their height, and their Euclidean distance from the rest of the detected peaks.

Supplementary Figure S1: The three input traces to the contraction identification algorithm: The black line denotes the original data trace on which the contractions should be identified. The other two are modified versions of that signal prepared ad-hoc: the blue dashed line is the smoothed version of this signal, and the yellow line is an enhanced version of the signal, with readily-detectable modulations in heartbeat peak amplitudes.

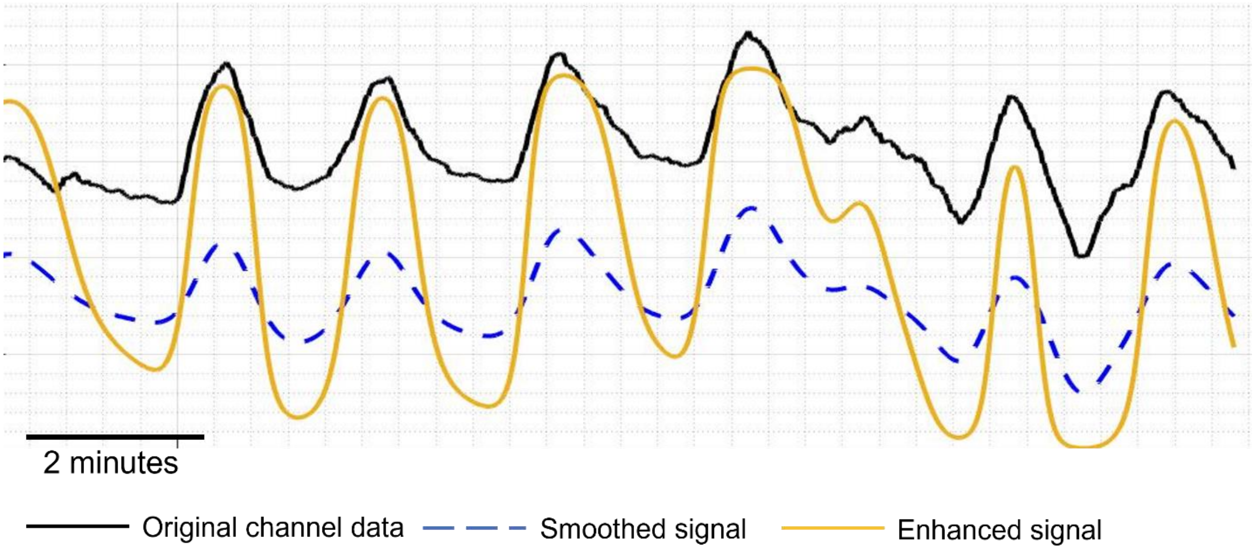

Supplementary Figure S2: A detailed flowchart of the main algorithmic steps of the Maternal Uterine Activity module (see chapter 2.3 in Materials and Methods section for more details) A. Pre-processing; B. Creation of surrogate uterine activity traces; C. Trace integration and fusion. For steps B and C the input data is the output of the previous step. Light-blue parallelograms refer to time series input/output; yellow parallelograms represent weight vectors input/output.

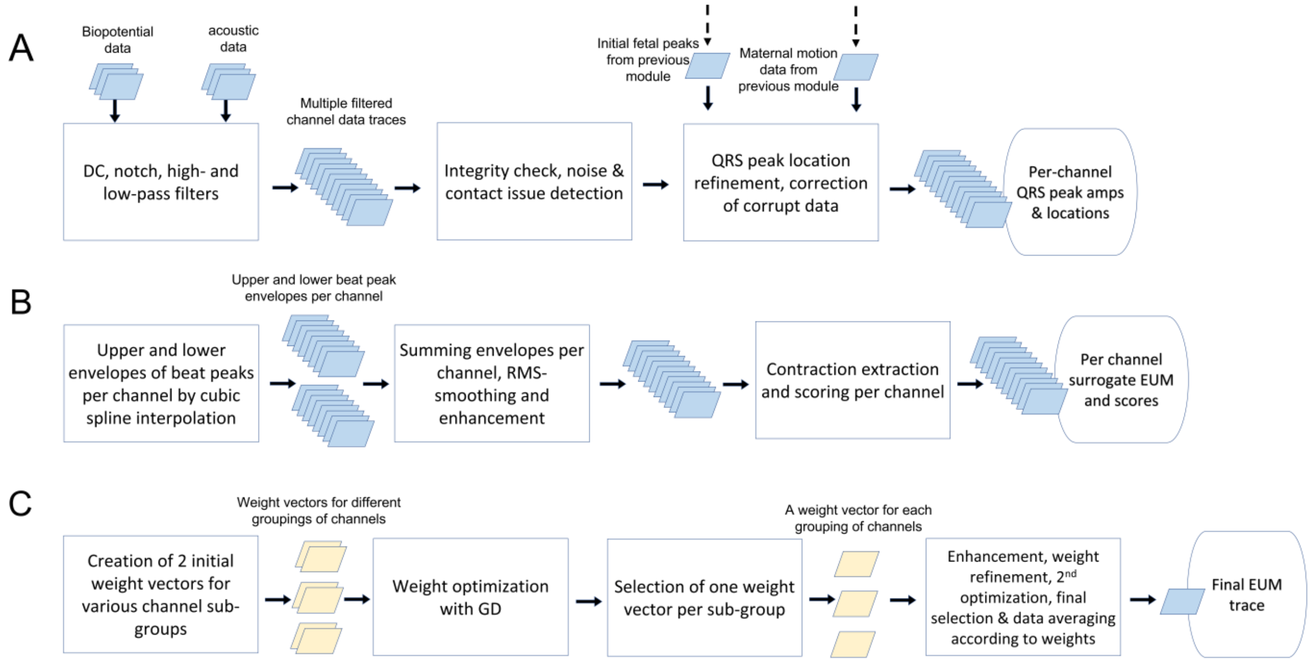

Supplementary Figure S3: An example of the weight evolution of two different channels and their corresponding traces, together with the final EUM signal for that session. Red indicates a low weight score, and green indicates a high weight score. For both channels, the first weight was created after 10 minutes – the first processed segment of the EUM signal lasted 10 minutes after which the first weight was calculated. For visualization purposes this weight was duplicated across the entire 10-minute segment. The following processed segments and corresponding weights were 1 minute long. **A.** In this channel the first weight was very low, and its corresponding trace (red) showed noisy and poor signal quality. Weights 20 to 30 (green) were created in ten 1-minute intervals during which the corresponding trace showed high quality signal. **B.** In this channel the first weight was high, and its corresponding trace demonstrated a high-quality signal. This quality then deteriorated at time segments 20-30 resulting in low weights created during this interval. **C.** The final EUM trace created for this session, to be used as reference showing how each channel and its corresponding weights affected the final result.

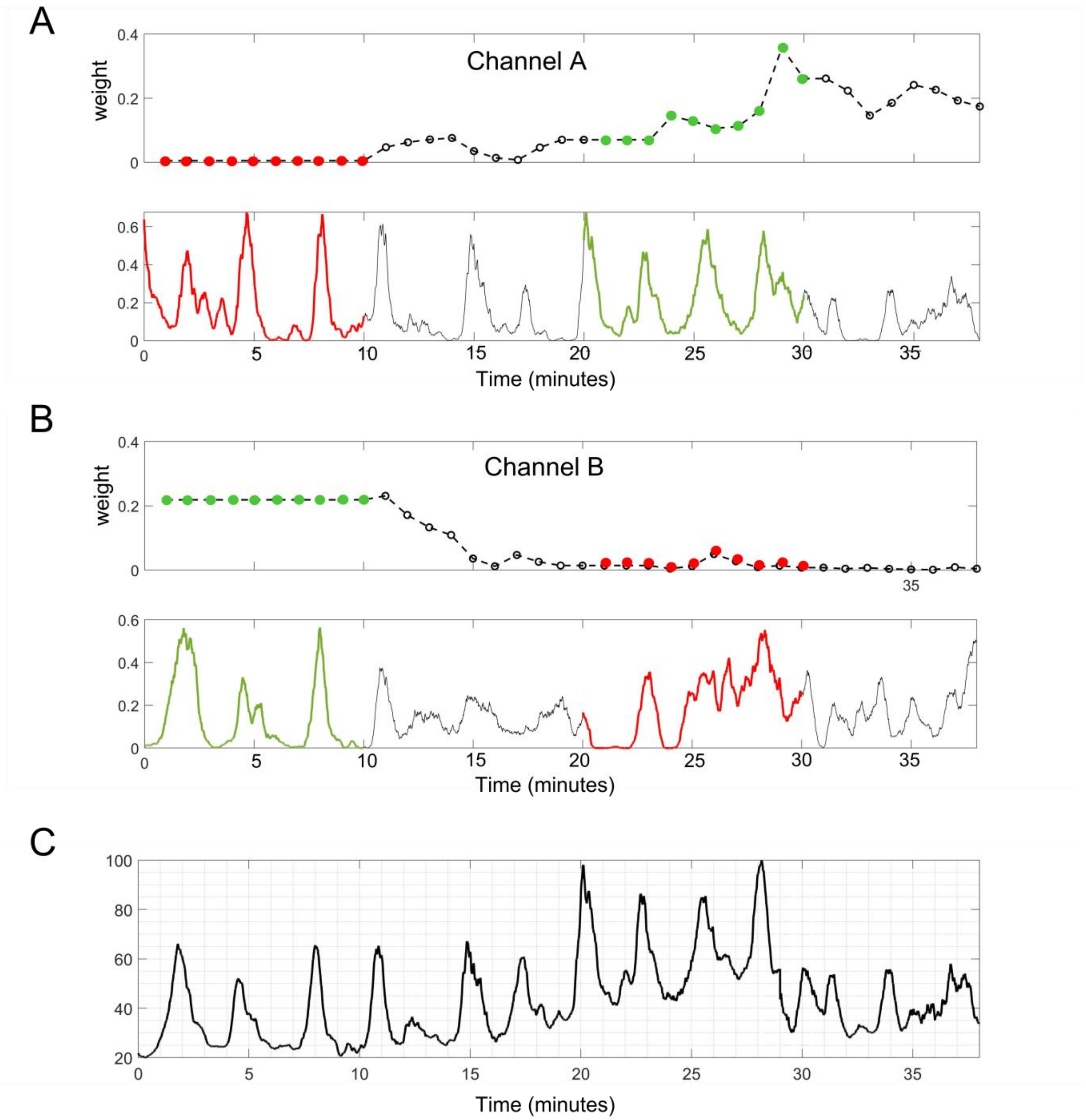
A
Channel A
weight
Time (minutes)
B
Channel B
weight
Time (minutes)
C
Time (minutes)

Supplementary Table S1: Intrapartum study demographics

Demographic Variables	All participants (n = 41)	Participants in 2-way comparision (n = 31)	Participants in 3-way comparison (n = 10)
**Age (years)**	26.7 ± 5.2 [18.0, 37.0]	27.5 ± 5.2 [18.0, 37.0]	24.1 ± 4.3 [19.0, 30.0]
**Gestational Age (weeks)**	38.8 ± 1.5 [34.0, 41.0]	38.5 ± 1.6 [34.0, 41.0]	39.6 ± 1.1 [38.0, 41.0]
**Body Mass Index (kg/m^2)**	29.6 ± 7.7 [18.1, 48.2]	30.0 ± 8.0 [18.1, 48.2]	28.3 ± 6.9 [21.1, 41.1]
**All data shown are mean ± standard deviation [minimum, maximum].**			

Supplementary Table S2: Antepartum study demographics

Demographic Variables for the Antepartum sessions	
**n**	16
**Age (years)**	33.2 ±7.1 [19, 42]
**Gestational Age (weeks)**	37.2 ± 2.2 [34, 40]
**Body Mass Index (kg/m^2)**	23.3 ± 3.5 [18.4, 30.1]
**All data shown are mean ± standard deviation [minimum, maximum].**	
